# Using co-design to develop an intervention to improve communication about the heart failure trajectory and end-of-life care

**DOI:** 10.1186/s12904-018-0340-2

**Published:** 2018-06-11

**Authors:** Lisa Hjelmfors, Anna Strömberg, Maria Friedrichsen, Anna Sandgren, Jan Mårtensson, Tiny Jaarsma

**Affiliations:** 1 Department of Social and Welfare Studies (ISV), Faculty of Health Sciences, Linköping University, 581 83 Linköping, Sweden; 2 Department of Medical and Health Sciences, Division of nursing, Faculty of Health Sciences, Linköping University, Linköping, Sweden; 3 Department of Cardiology, Linköping University, Linköping, Sweden; 4 Department of Health and Caring Sciences, Center for Collaborative Palliative Care, Linnaeus University, Växjö, Sweden; 5 Department of Nursing, School of Health Sciences, Jönköping University, Jönköping, Sweden

**Keywords:** Heart failure, Illness trajectory, End-of-life care, Communication, Co-design, User perspective

## Abstract

**Background:**

The aim of this paper was to describe the development of an intervention that is developed to improve communication about the heart failure (HF) trajectory and end-of-life care. We also present data that provides a first insight in specific areas of feasibility of the intervention.

**Methods:**

Co-design was used and patients, family members and health care professionals were constructive participants in the design process of the intervention. Feasibility of the intervention was tested in two areas; acceptability and limited efficacy.

**Results:**

Two communication tools were designed and evaluated; 1) a Question Prompt List (QPL) for patients and family members and 2) a communication course for professionals which was web -based with one face-to-face training day with simulation. Data on feasibility was collected with questionnaires that were developed for this study, from the 13 participants who completed the course (all nurses). They reported improved knowledge, confidence and skills to discuss the HF trajectory and end-of-life care. The QPL was evaluated to be a useful tool in communication with patients and family members.

**Conclusions:**

In a co-design process, future users identified the need for a QPL and a communication course. These communication tools can be used as a dual intervention to improve communication about the HF trajectory and end-of-life care. The QPL can help patients and families to ask questions about the HF trajectory and end-of-life care. The communication course can prepare the professionals to be knowledgeable, confident and skilled to discuss the questions in the QPL. Before the tools are ready for implementation in clinical practice, further studies testing the feasibility of the intervention are needed, including also patients and their families.

## Background

Heart Failure (HF) is a life threatening illness, often with a poor prognosis [1]. The HF trajectory is unique for each individual person, and it may include recurrent, unpredictable exacerbations, including decreased functional status [2–5]. Death from HF can be sudden, due to an ischemic event or electric instability of the heart, or it can be slow, due to episodes of decompensation or progressive organ failure [6]. Patients with HF also often have several comorbidities that can cause death [7]. Guidelines recommend health care professionals to have an open communication with patients and their families about the HF trajectory, including discussing their preferences for future care, acknowleding the risk of a sudden death, and the possibility of deactivation of implantable defibrillators in the end-of-life [8, 9]. A lack of insight in the HF trajectory, including disease progression and functional decline, can increase anxiety and uncertainty in patients and family members which may affect quality of life and also increase use of health care resources [10]. Additionally, if the HF trajectory is not adequately discussed, issues around end-of-life care tend to be addressed too late, possibly resulting in an unsatisfactory end-of-life care for both patients and families [11]. There seems to be a reluctance to talk about death and dying in clinical HF practice, and conversations focus mostly on disease management and less on preferences and goals of care [12]. Health care professionals are often hesitant to have these conversations as they are afraid of taking away hope and cause anxiety in patients and their families [12, 13].

However, patients with HF and their families would often welcome conversations about the HF trajectory and end-of-life care, but do not always know how to initiate the discussion with health care professionals [12]. Several studies [12, 14–17] have described the needs for improved communication about the HF trajectory and end-of-life care in HF care. However, there is limited knowledge on the most appropriate communication strategies that meet the specific needs of patients with HF, their families and the health care professionals. Interventions to improve communication should be developed in accordance with the future users’ needs and preferences to increase their usefulness. During the last decades, methods in which patients, family members and health care professionals are involved in the design of health care services have become more common [18]. Examples of such methods are Experienced-based design, Co-design and experienced-based co-design, in which staff and patients (or other service users) co-design services and/or care pathways, together in partnership [19]. A core methodological approach in these methods is involving the experiences of future users in the design process [19], focusing on both understanding and improving a person’s experiences of a product/service as well as the product/service itself [20, 21]. Key benefits of co-design methods in development studies include the possibility to engage and empower patients, family members, and health care professionals to become active participants in the development of services that they will use in the future [19]. The aim of this paper was to describe the development of an intervention that is developed to improve communication about the HF trajectory and end-of-life care. We also present data that provides a first insight in specific areas of feasibility of the intervention.

## Methods

### Co-design

**Fig. 1:**
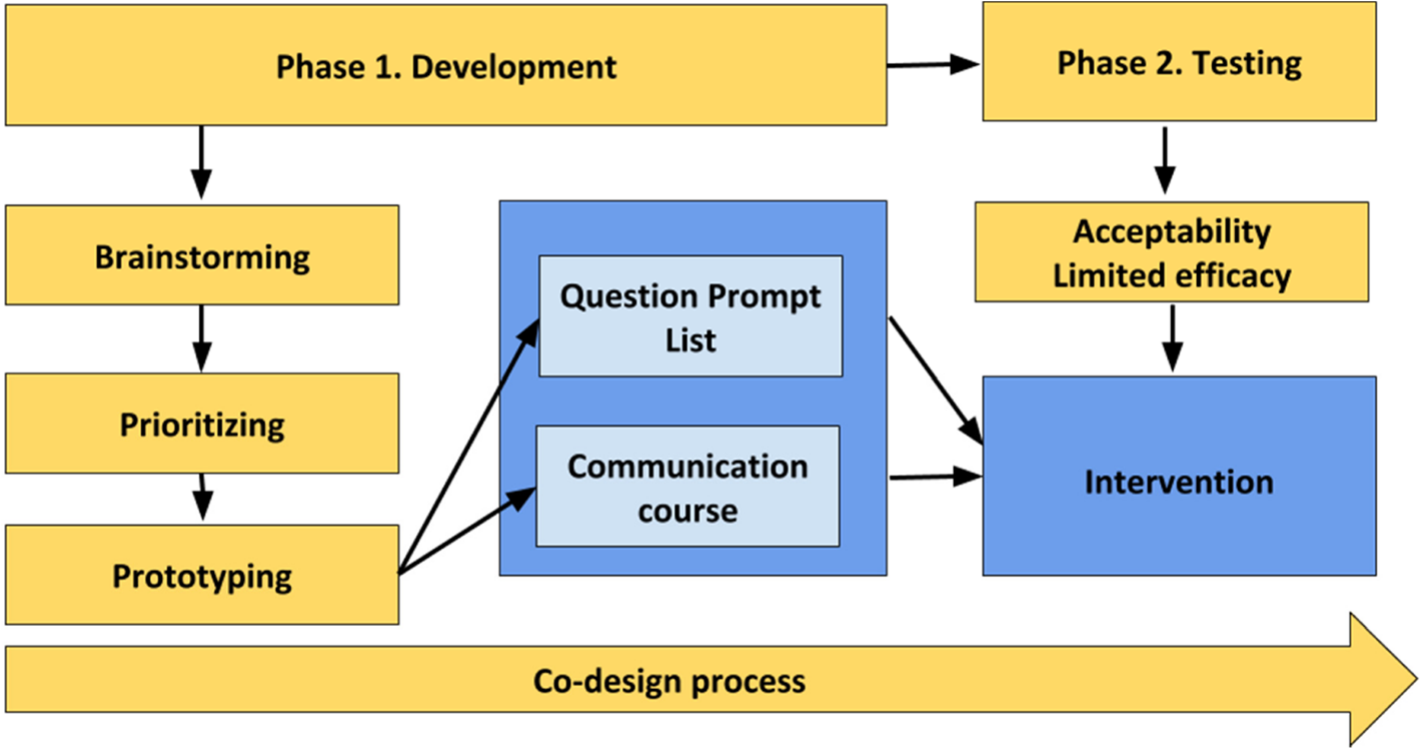
The co-design process used in the study included phase 1, developing the intervention, and phase 2, testing the acceptability and limited efficacy of the intervention

**Fig. 2:**
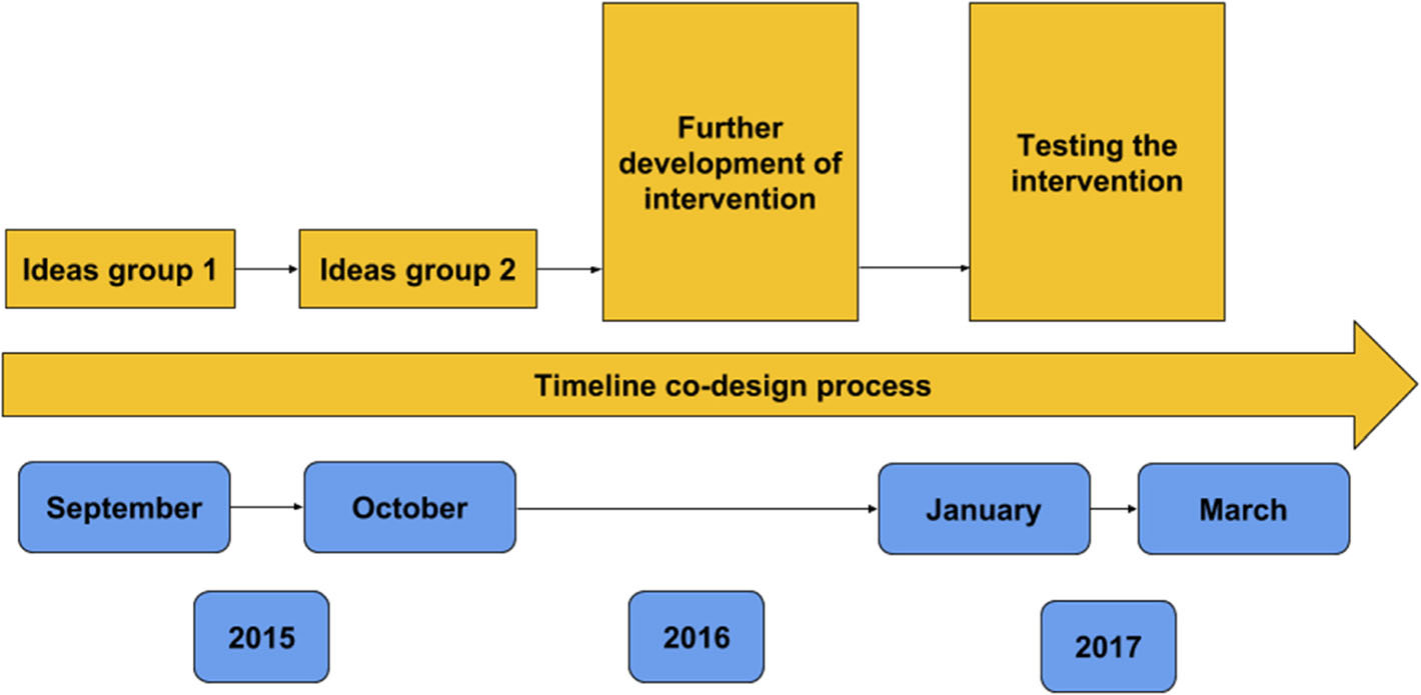
Timeline of the co-design process including two ideas groups with patients, family members and health care professionals, participating in the development of the intervention. During 2016 the intervention was further developed by the research group and in 2017, acceptability and limited efficacy of the intervention was tested

Patients with HF, family members and health care professionals were invited to be constructive participants in the design process of the intervention. After that, health care professionals participated in a first feasibility testing of the intervention. The intervention was developed and tested in two phases, (phase 1: Development, and phase 2: Testing, Fig. 1), which took place from September 2015 to March 2017 (Fig. 2). In the co-design process, the concepts “Ideas groups” and “Prototyping” were used as described in the Health Service Co-design toolkit [20]. Ideas groups is a tool that can be used to brainstorm ideas for improvement and ways of implementing them in clinical practice. Prototyping can be used to test new products to see if they will work and it is a useful way to engage and stimulate creativity among the stakeholders taking part in ideas groups [20].

### Phase 1- developing the intervention

First, stakeholders came together in ideas groups at two different time points to brainstorm improvement ideas and ways to implement them in clinical practice. The goal with the ideas groups was described to the participants as to develop communication tools/support to improve communication between patients, family members and health care professionals about the HF trajectory and end-of-life care. At the first ideas group, the goal was to discuss ideas that could improve communication, and at the second ideas group, the goal was to further develop and discuss the ideas that were suggested in the first ideas group. Patients with HF, their family members, and health care professionals, were invited to participate in the ideas groups. A HF nurse in an outpatient HF clinic identified suitable patients and provided their names, ages, and information on their New York Heart Association Functional Classification (NYHA class). The first author sent a study invitation letter to the patients and then called them a few days later to ask if they were interested to participate in the study, with or without a family member. The researchers used their own network of physicians and nurses with expertise in HF care and palliative care, and invited them by email to participate in the study. The patients (*n* = 9) who agreed to participate were in NYHA class I-III and had a mean age of 75 years. The family members (*n* = 2) who agreed to participate in the study had a mean age of 70 years. None of the patients were recently diagnosed, most of them had lived with HF for several years. Some of them had medical devices such as an Implantable Cardioverter Defibrillator (ICD), or Cardiac Resynchronisation Therapy (CRT). The professionals (1 physician and 8 nurses, mean age 50 years) had extensive experience of working in HF care or palliative care. The researchers (*n* = 5) were 4 nurses with experience in co-design, HF care and palliative care, and 1 behavioural scientist with experience in medical education. The researchers led the ideas groups, facilitated and summarized the discussions, took field notes and made audio recordings.

### Phase 2- testing the intervention

The intervention was tested in phase 2 of the study. The testing focused on two areas of feasibility; acceptability and limited efficacy of the intervention [22]. Acceptability refers to the participants’ satisfaction with, and reactions to the intervention. The limited efficacy testing evaluated the intervention’s potential of being successful among the intended users, meaning that the intervention can have an impact on knowledge, skills and confidence of health care professionals in the future [22]. To measure acceptability and limited efficacy in the testing of the intervention, data were collected pre-post intervention, using questionnaires that were developed for this study. The questionnaire that measured the participants’ satisfaction with, and reactions to the intervention (acceptability), included open- and closed ended questions and statements using a 4-point Likert scale (1 = strongly agree to 4 = strongly disagree). The questionnaire that measured the participants’ knowledge, confidence and skills to discuss the HF trajectory and end-of-life care (limited efficacy), included statements using a 4-point Likert scale (1 = strongly agree to 4 = strongly disagree). A 2-week test-retest reliability of the questionnaire evaluating limited efficacy, was assessed in a group (*N* = 13) of cardiac health care professionals before it being used in the present study. The test-retest reported the kappa values of the 50 items in the questionnaire. Thirty-four items had a good to moderate agreement, 0.41–0.80, and 16 items had a fair agreement 0.20–0.40 [23].

To test the intervention (the Question Prompt List, QPL and the course) an invitation letter was sent out to HF clinics in all regions in Sweden. All cardiac professionals who were interested in the content of the course were eligible to participate. Twenty-one cardiac professionals working in a clinical setting signed up for the course, 3 physicians and 19 nurses. Before the course started and during the first weeks of the course, 6 participants (all nurses) withdrew due to changes in their work situation or due to private matters. Sixteen participants (Table 1) followed web-lectures, completed the 2 assignments (Table 2) and attended the training day. None of the 3 physicians completed task 3 (Table 2). The reasons for this is not known.

**Table 1:** Course content and teaching methods

Three individual tasks (online)	1) The participants reflect on when they think is the best time to discuss about the HF trajectory with a patient for the first time and give reasons for their choices. 2) The participants familiarize themselves with the QPL and reflect on their own knowledge and ability to converse with the patient and family about the different questions in the QPL. 3) The participants use the QPL in their clinical work with one patient and/or one family member.
Lectures (online)	1) Why do we need to talk about the HF trajectory and end-of-life care? -15 min 2) Discussing the HF trajectory and end-of-life care - Who, what and when? -25 min 3) Patients with heart failure pacemaker or ICD- how to end the treatment − 15 min 4) Patients’ experiences of, and preferences for communication about the HF trajectory − 10 min 5) The existential conversation − 15 min 6) End-of-life care communication in HF -20 min 7) Communication with the family members − 10 min
Group discussions (training day)	Participants are divided into small groups with a group leader. The participants reflect on what questions in the QPL they perceived as easy or difficult to discuss. Through group discussions, participants can learn from each other’s experiences.
Simulation (training day)	Two actors simulate the role of a patients with HF who want to discuss questions in the QPL and the participants act as health care professionals. The participants take turns to simulate a family member in the conversations and take part in the discussion afterwards.

*QPL* Question Prompt List, *ICD* Implantable cardioverter defibrillator, *HF* Heart Failure

**Table 2:** Background characteristics of the course participants

*N* = 16	
Sex (*N*, %)	
Females	16 (100%)
Age (years, mean)	24–57, 39
Occupation (*n*, %)	
Physician	3 (19%)
Nurse	13 (81%)
Workplace	
Hospital	13 (81%)
Public health care centre	2 (13%)
Hospitalized home care	1 (6%)
Specialist education^a^	
Yes	10 (60%)
Time working with HF patients (years, mean)	0–25, 9

*HF* Heart Failure. ^a^Physicians- general or internal medicine, nurses- cardiology or public health

**Table 3:** Acceptability: Participants’ satisfaction with, and reactions to the course (*N* = 13)

Statements	Strongly agree/agree
Lecture 1- “Why do we have to talk about the HF trajectory and end-of-life care with patients and their families?” was a worthwhile lecture	13 (100%)
Lecture 2- “Discussing the HF trajectory and end-of-life care” was a worthwhile lecture	13 (100%)
Lecture 3- “Patients’ experiences and preferences of discussing the prognosis” was a worthwhile lecture	13 (100%)
Lecture 4- “Patients with pacemaker and/or ICD- decisions on determination of treatment” was a worthwhile lecture	13 (100%)
Lecture 5- “Enabling existential communication” was a worthwhile lecture	13 (100%)
Lecture 6- “Palliative communication in HF care” was a worthwhile lecture	13 (100%)
Lecture 7- “Communication with family members” was a worthwhile lecture^a^	12 (92%)
Task 1 was worthwhile to do	11 (85%)
Task 2 was worthwhile to do	12 (92%)
Task 3 was worthwhile to do	11 (85%)
The group discussions during the training day was worthwhile	12 (92%)
The Question Prompt List will be a useful tool in my future communications with patients and their families	11 (85%)
The course literature have contributed to my learning	11 (85%)
The provided web -sites on the course site was worthwhile to watch	12 (92%)
It was good that the course was web-based	13 (100%)
The web-site of the course worked well	13 (100%)

^a^One participants could not view this lecture and therefore did not agree with the statement

### Data analysis

Data from the ideas groups (field notes and audio recordings) and data from the open-ended questions in the questionnaires were evaluated, discussed and summarized in the research group. As the aim was to identify improvement ideas expressed by the participants, and evaluate the intervention, the data were summarized without an in-depth qualitative analysis. Descriptive statistics were used to describe the participants’ background characteristics. Frequencies and proportions were used to describe the outcomes in the questionnaires.

## Results

### Phase 1- developing the intervention

#### Brainstorming

Ideas group 1 started with a short presentation by the research group, introducing some background information of life-limiting nature of HF. After that, during a brainstorming session, participants were encouraged to share their experiences of bad or good communication situations in health care. Some patients described a lack of information of their HF from the health care, and emphasised the possibility to get repeated information at different time points. It was considered a problem to remember information as well. The patients also believed that bad communication could depend on the patients themselves, as many might have difficulties to pose questions to the health care professional. Family members described a lack of information on the seriousness of the illness and a wish to receive the same information as the patient. Some kind of a communication tool that could help patients and family members to ask questions would be helpful. Meeting other patients in a group discussion with a nurse as a facilitator of discussion, was suggested as one way to enhance communication.

In the brainstorming, the health care professionals described that it was difficult in clinical practice to know what had already been discussed with patients/family members. Continuity in care was considered important, and a HF nurse could have a crucial role to play in communication, as the nurse is the one that most often meets the patients/family members. Communication training for professionals as a way to improve communication was also suggested.

After hearing and discussing the participants’ experiences of communication and various ideas of communication tools/support, the researchers also presented communication interventions that had previously been used in cancer care, to be used as examples to discuss and draw inspiration from [24–26].

In summary, suggested ideas for interventions for the patient and their family members included:

- patient meetings in groups in which patients can support each other
- family conversations in which patients, family members and all health care professionals involved in the care can meet and discuss the future
- a list for patients and family members with questions about the HF trajectory and end-of-life care to be used as a communication tool.

Suggested ideas for interventions for health care professionals included:

- communication training in groups
- individual web-based education in communication
- role play or a film that provides a good example of a conversation
- simulation training of conversations about the HF trajectory and end-of-life care.

An important comment from the participants was that instead of focussing on one group of users (e.g. only make a tool for patients) both an intervention for the patients/family members and an intervention for the professionals could be useful to improve communication.

#### Prioritizing

The participants voted for the two ideas that came up in the brainstorming that they thought could make the biggest difference in improving communication. The idea of a list with questions about the HF trajectory and end-of-life care for patients and family members, and communication training for health care professionals were the two most popular ideas.

#### Prototyping

Two months after the first ideas group, the participants met again in an ideas group to further discuss and develop the list of questions and the communication training into prototypes. The participants brainstormed useful questions that could be included in the list and discussed useful wording and language. Following the second ideas group, the prototype of the list was further refined by the researchers, based on the suggestions from the ideas groups and relevant literature. The prototype of the list was sent to the ideas group participants for evaluation. The participants were asked to comment on the layout as well as the content. The majority of the participants found the list to be in concordance with their suggestions.

Further on, the communication training was refined into a communication course prototype by the researchers, based on the suggestions from the ideas groups. Suggested content for the course included, for example, theoretical knowledge about communication, as well as practical training using simulated conversations and role play.

#### The intervention

When finalizing the prototypes, the research group decided that the two prototypes, the list of questions and the communication training, should be combined into a dual component intervention and be used together. The list of questions will from now on be referred to as the QPL and the communication training will be referred to as the communication course.

#### The question prompt list

The QPL was designed to stimulate and facilitate patient and family member communication about the HF trajectory and end-of-life care with a health care professional. The QPL is a 7-page A4 booklet, containing 45 questions grouped into the topics: 1) Heart failure and what to expect in the future 2) Help and support at deterioration 3) End-of-life care issues 4) Additional questions for the family members 5) Additional questions for the person with an ICD/CRT/PM. The purpose of the QPL is to function as an aid for patients and family members at clinical appointments, and encourage them to ask questions that are relevant to them about the HF trajectory and end-of-life care. They can also add more questions that are not in the list. The QPL is expected to function as a communication tool to help the patients and families to be more involved in the care.

#### The communication course

The communication course is web-based with one face-to-face practical training day, designed to be applicable for health care professionals who work in cardiology. Nurses in cardiology and palliative care and a behavioural scientist act as course leaders. Participants take the course at their own convenience. It is based on adult learning principles using self-directed learning [27] and includes several teaching methods (Table 2).

The learning goals of the course are for the participants to expand their knowledge about communication about the HF trajectory and end-of-life care, and to improve on their own knowledge, confidence and skills in communications. The QPL is an important tool in the course and the participants are expected to learn to use the QPL as a communication tool in the course and to gain the needed knowledge, confidence and skills required to discuss the questions in the QPL with patients and family members. Another important part of the course is the practical training with simulated patients (actors) and family members (volunteer course participants), where the participants practice using the QPL in a simulated conversation. Constructive feedback on their performance is provided by course leaders, the simulated patients and family members, as well as the other participants in the course.

### Phase 2, testing the intervention

#### Acceptability: Participants’ satisfaction with, and reactions to the course

The participants were asked to evaluate the course, using close-ended (Table 3) and open-ended questions. They described that they took the course simultaneously with their clinical jobs and reported to have spent 10–30 h on the course. The participants expressed that the course had helped them to reflect on their professional role in discussions about the HF trajectory and end-of-life care, which made them more confident in discussions without worrying about providing the “correct” answers to patients and family members. All the participants evaluated the seven lectures to be of importance for their learning and most evaluated the three individual tasks worthwhile to do. They reported that meeting colleagues to discuss and learn from each other as well as practicing having conversations with simulated patients and family members during the training day was valuable. Overall, the participants were satisfied with the content of the course and how it was delivered, but some (*n* = 5) would have preferred to have one additional face-to-face training day as a follow-up. The majority of the participants had no technical problems with the web -site, but 3 participants reported difficulties to view one of the lectures. All participants would recommend the course to a colleague. After completing task 3 (using the QPL in clinical practice), most (*n* = 11) reported that the QPL would be useful in future clinical conversations, but they thought that the QPL could be shortened.

#### Limited efficacy

**Fig. 3:**
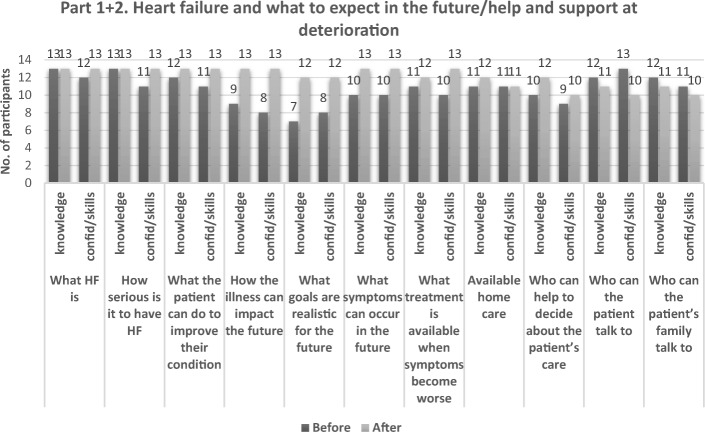
The number of participants agreeing (strongly agree/agree) to have knowledge/confidence/skills to discuss questions in part 1–2 of the QPL, before and after the course, *N* = 13. Abbreviations: QPL Question Prompt List

**Fig. 4:**
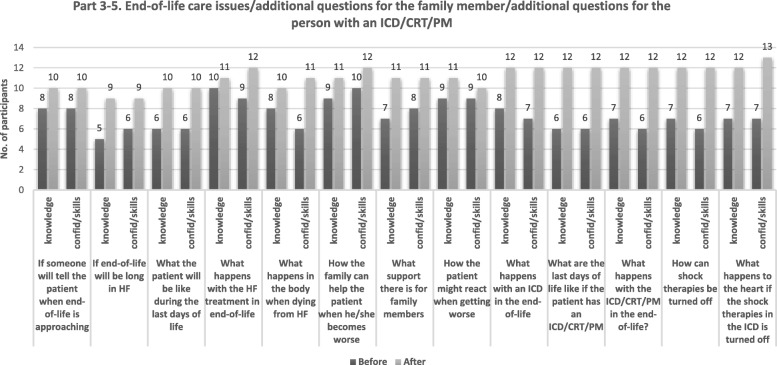
The number of participants agreeing (strongly agree/agree) to have knowledge/confidence/skills to discuss questions in part 3–5 of the QPL, before and after the course, N = 13. Abbreviations: ICD Implantable Cardioverter Defibrillator, CRT Cardiac Resynchronisation Therapy, PM Pacemaker, QPL Question Prompt List

In a pre-post-test, questions from the QPL that would mirror the learning goals of the course were included, assessing the participants’ self-reported knowledge, confidence and skills for each question. For the majority of the participants, on most of the questions their knowledge, confidence and skills was reported to have increased after the course (Figs. 3 and 4). However, the participants reported after the course to have less knowledge, confidence and skills to discuss two of the questions in part 2 of the questionnaire, concerning who the patient/family member can talk to about the things that worry them.

## Discussion

To our knowledge, this is the first study to use a co-design method and invite the future users in the development of an intervention aimed to improve communication about the HF trajectory and end-of-life care in HF care, and test the acceptability and limited efficacy of the intervention. In co-design, the ‘co’ is pivotal, emphasizing a collaborative effort by patients, families, professionals and the researchers facilitating the process [28]. The co-design method made it possible for patients, their families, and health care professionals to share their experiences. Through a partnership with the researchers, the patients and their families contributed to the design and development of the intervention, which ensured that the intervention was based on their needs [20]. In the co-design process, two ideas were suggested and developed into prototypes, a QPL and a communication course that formed the intervention. To evaluate if the intervention could be useful in clinical practice, the two areas of feasibility that were found most important at this stage of the study were tested; acceptability and limited efficacy [22]. The results show that the course was well received by all the nurses who finalized the course. The QPL was tested by nurses in clinical practice with positive results and was reported to be a valuable tool in communication, helping the patients and the family members to ask questions about the HF trajectory and end-of-life care. Similar results have been found in a recent extensive literature review on Question Prompt Lists in cancer care, reporting that most patients find using a QPL useful to frame questions and enhance the consultation [29]. A study from 2001 [30] described the use of a checklist with questions to improve patient education in cardiology and in the evaluation of the long-term efficacy of the checklist, it was concluded that the it could be a useful tool for preparing the patient for the visit to the cardiologist [31]. Our QPL showed similar results, but it is different as it is supposed to be used in discussions between patients, their families and several cardiac professionals, not only by a cardiologist in a doctor-patient communication.

The participants’ perceived knowledge, confidence and skills to discuss the HF trajectory and end-of-life care in HF care, increased after the course, with the biggest changes concerning issues around ICD/CRT. This is not surprising as these are areas that are difficult to discuss with patients and family members [32], and health care professionals might have little knowledge about them. The participants reported decreased knowledge, confidence and skills in two questions that concerned who the patient or the patient’s family can talk to if they feel a need for help and support. This, however, does not necessarily mean that the participants’ knowledge, confidence and skills actually decreased. Rather, during the course, they might have become more aware of the fact that there are more aspects related to certain topics then they originally thought. The participants might have become aware of their lack of competence, which can be an important first step towards gaining the needed knowledge, confidence and skills to discuss these topics [33]. This is in line with the adult learning principles that aim to facilitate a problem-solving process for the learning individual by identifying a lack of knowledge in order to gain new knowledge [34].

We chose to assess not only the participants’ knowledge, but also their confidence and skills to discuss the HF trajectory and end-of-life care, as our previous studies found that even if a person has knowledge to discuss those topics, it does not mean that they have the confidence and skills to actually take on the conversation [13].

To further improve the intervention, other areas need to be tested, such as implementation and demand [22]. These areas of feasibility can be explored in a later phase.

There are some important limitations to consider in this study. First, although the results of the testing are promising, it has to be recognised that due to the limited number of participants and the lack of statistical analyses, more information is needed to draw definite conclusions about the efficacy of the course. Although we are further developing the materials, we find it important to share our results in this stage so readers can be inspired to use or further develop the intervention to improve their care to HF patients. Secondly, input from family members was limited as only two family members participated in the development of the intervention. Thirdly, as we only send an evaluation form to those who completed the final task, we missed specific data on the satisfaction from the three physicians, who did not complete task 3, which limits the possibility to state anything about the physicians’ perceptions of the intervention, and also raises the questions if the final task was useful for training physicians. The lack of physicians must also be seen as limitation of the generalizability of the intervention to non-nurse professionals. Preferably, physicians from both general care and cardiology should have participated in all parts of the study in order for it to be more effective and strengthen the validity.

In addition, the data were self-reported and not based on direct observations of the participants’ knowledge, confidence and skills, and, due to the small sample size, we could not calculate for any significate changes pre- and post the intervention, but only look at positive trends in the data. It is also important to consider that no men participated in the testing of the intervention and that there might be a potential selection bias if only professionals that were already motivated to learn more about communication about the HF trajectory and end-of-life care, are represented in the sample. Most participants attending the course already had some knowledge, confidence and skills to communicate about the HF trajectory and end-of-life care. At the same time, the majority of the participants also reported having learned more during the course.

To evaluate knowledge, confidence and skills related to the QPL, a questionnaire was developed especially for this study instead of using a general one. However, the results from the questionnaire need to be interpreted with caution, as the questionnaire was only psychometrically tested in a limited extent.

Additionally, it also should be noted that the patients involved in the development of the intervention were not the ones with the most advanced HF; hence this intervention was developed with HF patients at earlier stages of the illness and their ideas for effective interventions may differ from those with more advanced HF.

One important advantage in the co-design process was the diverse sample of participants that participated in both the development and the testing of the intervention. This strengthens our findings and entails that the intervention is based on the needs of future users, which is crucial in order for the it to be useful [35]. Many patients, families and health care professionals are interested in taking part in development studies as they want to see a change and improvement in practice [19]. In this study, some participants contributed more and were more creative than others, but everybody showed an interest in the co-design process and contributed to some extent. The researchers took on a facilitator’s role in the design process, bringing knowledge and expertise from their own professional backgrounds, thus guiding and inspiring the design process [21].

## Conclusions

We have developed and evaluated an intervention containg a QPL for patients and family members and a communication course for cardiac health care professionals using a co-design approach including those groups representing the future users in the process. This intervention can be a first step towards improving communication about the HF trajectory and end-of-life care in HF care. The intervention has a dual approach to communication, where the QPL can help patients and family members to initiate discussions about the HF trajectory and end-of-life care, whereas the communication course can help professionals to gain the knowledge, confidence and skills required to discuss the questions raised by patients and their families. Before the tools are ready for implementation in clinical practice, further studies testing the feasibility of the intervention are needed, including also patients and their families.

## Abbreviations

CRT: Cardiac Resynchronisation Therapy
HF: Heart Failure
ICD: Implantable Cardioverter Defibrillator
NYHA class: New York Heart Association functional classification
PM: Pacemaker
QPL: Question Prompt List
